# Hiding in plain sight: Cryptic enemies are found on cochineal (Hemiptera: Dactylopiidae), a scale insect of economic and cultural significance

**DOI:** 10.1002/ece3.9151

**Published:** 2022-07-31

**Authors:** Suzanne E. Kelly, Wendy Moore, W. Eugene Hall, Martha S. Hunter

**Affiliations:** ^1^ Department of Entomology The University of Arizona Tucson Arizona USA

**Keywords:** biological control, cactus pest, Chalcidoidea, Coccinellidae, parasitic wasp, pest cactus

## Abstract

Cochineal is the common name for cactus‐feeding scale insects in the Dactylopiidae. These ruby‐red insects include the domesticated dye insect *Dactylopius coccus*. *Dactylopius coccus* and congeners have been introduced around the world, some accidentally, to become pests of prickly pear cactus species (*Opuntia*), and some intentionally, for dye production or biological control of pest *Opuntia.* In the northern Sonoran Desert (Tucson, AZ, USA), we studied the enemy complex of *D. opuntiae* and *D. confusus* on *Opuntia* and characterized two cryptic enemies, a coccinellid beetle predator and a parasitoid wasp. (1) *Hyperaspis* sp*.* The coccinellid predator *Hyperaspis trifurcata* shares a niche with a similar, typically all‐black beetle. Morphological data, crossing tests, and phylogenetic results showed the black beetle to be a distinct, undescribed species in the genus *Hyperaspis*, with a rare spotted phenotype that is similar in appearance to *H. trifurcata.* Crossing tests among black and spotted forms showed the spotted morph is inherited as a single‐locus dominant allele. (2) *Formicencyrtus thoreauini.* Rearing of this ant‐like parasitoid wasp (Encyrtidae) in pure culture of *D. opuntiae* showed it to be a semi‐gregarious primary parasitoid of cochineal*.* To our knowledge, this is the first confirmed instance of a cochineal parasitoid. Observations of development show early instar larvae keep their posterior end within the egg chorion, attached to an aeroscopic plate with a connection to the cochineal body wall. Late instar larvae are eventually surrounded by a membrane, likely of larval origin. Wasps then pupate in a dry air‐filled chamber within the desiccated scale remains before chewing out as an adult. Both *Hyperaspis* sp. and *F. thoreauini* may have restricted distributions. *Hyperaspis* sp. does not appear to be a member of the cochineal community in Mexico or Texas, and scant records suggest *F. thoreauini* may also be restricted to the Southwestern USA.

## Graphical abstract

We characterize two cryptic enemies of the community of the scale insect cochineal in Tucson, Arizona, USA in the Sonoran Desert. A typically all black undescribed *Hyperaspis* sp. shares a niche with *Hyperaspis trifurcata*. We also show that a micropterous ant‐mimic wasp is a semi‐gregarious primary parasitoid of cochineal, to our knowledge the first parasitoid of cochineal described.

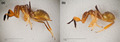

## 1 INTRODUCTION

Specialist enemies that attack cochineal are notable because of the long history of human fascination with their prey. The iconic bright red scale insects, clothed in cottony wax, include 11 species in the monogeneric family Dactylopiidae (García Morales et al., 2016). Long before European arrival to the New World, the Aztecs domesticated *Dactylopius coccus* Costa for its red hemolymph, the source of a brilliant red dye (Greenfield, 2006), making this species one of only a few domesticated insects, along with the honeybee and silk moth. In the early 19th century, over 3 million pounds of dried insects were exported from Mexico in a single year (1802), before alternative red dyes and overseas production caused the profitability of the local industry to decline (Hunter et al., 1912). While the prickly pear cactus species in the genus *Opuntia* and all cochineal species are native to the Americas, both host plants and cochineal have been widely introduced to warm arid climates around the world in the last 200 years, some *Dactylopius* for cochineal dye production (often without regard to species identification) and some *Opuntia* for fodder, fruit production, and erosion control.

*Opuntia* and cochineal introductions have long spurred conflicts of interest since prickly pear is considered a valuable plant in the Middle East and North Africa. For example, *Opuntia* is a forage and fruit crop in Morocco and a dry‐adapted hedgerow species of cultural significance in Israel (Bouharroud et al., 2019; Mendel et al., 2020; Paterson et al., 2021; Paterson & Witt, 2022; Spodek et al., 2013). A recent invasion of *Dactylopius opuntiae* (Cockerell) in Israel, Lebanon, and Morocco caused significant cactus mortality in plantations and landscapes (Bouharroud et al., 2019; Spodek et al., 2013). In Israel, a biological control program for *D. opuntiae* was launched and two specialist predators, a coccinellid beetle, *Hyperaspis trifurcata* Schaeffer, and a chamaemyiid fly, *Leucopis bellula* Williston, were introduced (Mendel et al., 2020). Conversely, prickly pear species are invasive plant pests of rangeland in several areas, including Kenya, Southern Africa, India, and Australia. In these locations, cochineal species have been important classical biological control agents for the cactus (Annecke & Moran, 1978; Novoa et al., 2019; Paterson et al., 2021; Paterson & Witt, 2022; Witt et al., 2020). In parts of Africa, the establishment of introduced predators of cochineal elsewhere on the continent poses a potential threat to the management of prickly pear (Paterson & Witt, 2022).

The diversity of cochineal and its specialist natural enemies span North and South America (Portillo, 2009). In the USA, there are a few species in the Southwest, especially in the southern regions of Texas through to California (Badii & Flores, 2001). In Tucson, in southern Arizona, we studied the shared enemies of two local cochineal species, *D. opuntiae* and *Dactylopius confusus* (Cockerell). Both species have been recorded as native to the study area (Mann, 1969), but are generally found on different *Opuntia* species. *Dactylopius confusus* is most often found on *Opuntia engelmanii* Salm‐Dyck, a native cactus to the southwestern USA. However, we found richer communities of predators and their parasitoids associated with the higher densities of *D. opuntiae* found on a spineless “Burbank hybrid” of *Opuntia ficus‐indica* (L.) Mill that is common in suburban plantings in the city of Tucson (Anderson & Olsen, 2015)*. Opuntia ficus‐indica* is native to central Mexico (Griffith, 2004). Among the natural enemies we observed, we found broad overlap with several key predator specialists described in communities throughout Mexico, and Texas. In all locations, four major predator species predominate: a coccinellid, *Hyperapsis trifurcata*, an unusual predatory caterpillar, *Laetilia coccidivora* Comstock, a chamaemyiid fly, *Leucopis bellula*, and one or more brown lacewing species in the genus *Sympherobius* (Gilreath & Smith, 1988; Vanegas‐Rico et al., 2010).

**FIGURE 1:**
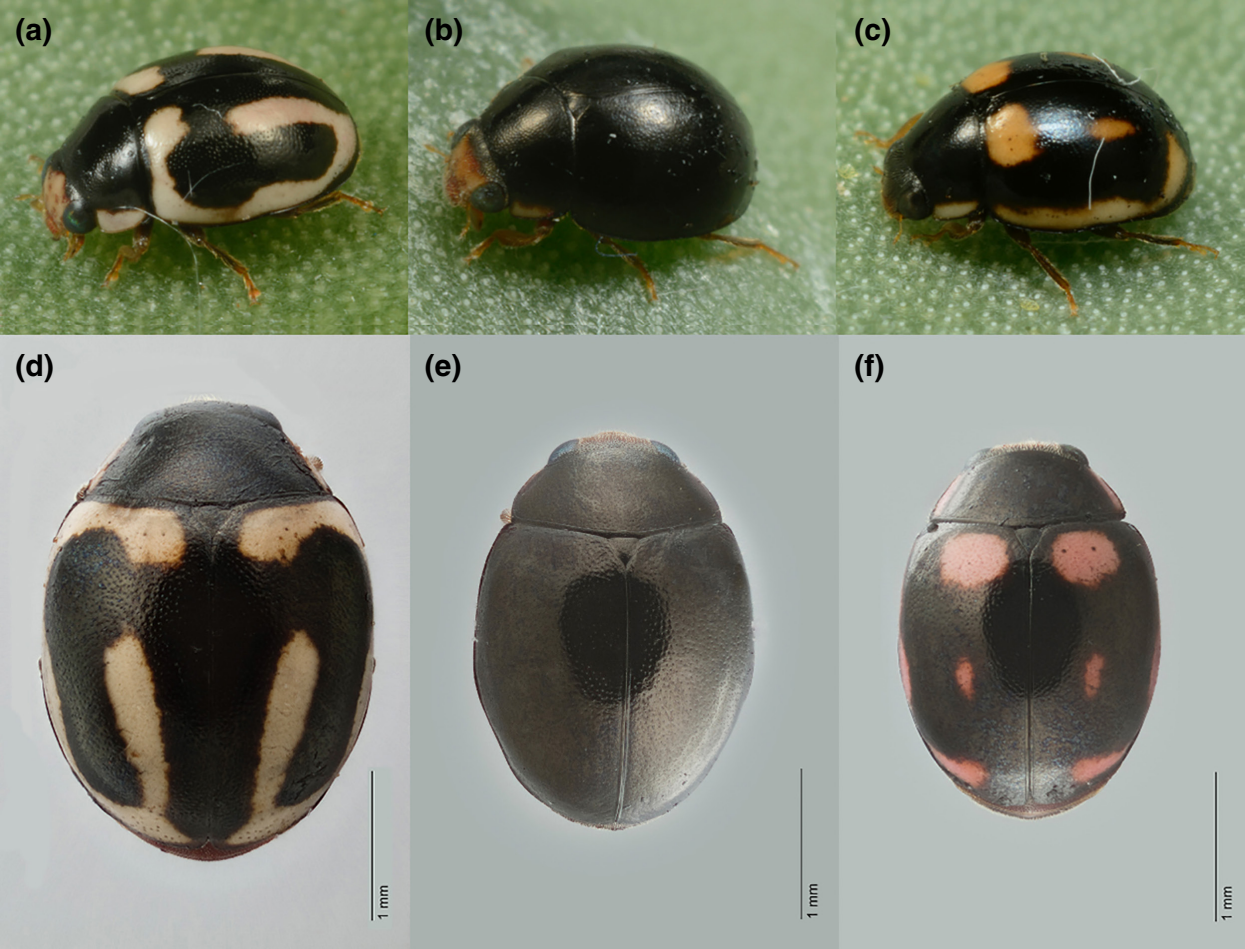
Phenotypic diversity of coccinellid beetles on *Dactylopius opuntiae* in southern Arizona, USA. a–c show the three beetle phenotypes in life, while d–f are higher resolution images of specimens shown at the same scale. (a and d) *Hyperaspis trifurcata*. (b and e) *Hyperaspis* sp. (c and f) A rare color morph of *Hyperaspis* sp.

In Arizona, we found additional cochineal natural enemies that were not, to our knowledge, referenced in the ecological literature: coccinellid beetles that were different in appearance from *H. trifurcata*, and a parasitoid wasp, *Formicencyrtus thoreauini* Girault. Here, we characterize two coccinellid beetle phenotypes, eventually, both confirmed as conspecific morphs of an undescribed *Hyperaspis* sp., with reference to the third common phenotype determined to be *H. trifurcata*. The two uncharacterized morphs included a common beetle with entirely black elytra of similar size to *H. trifurcata*, and a rare spotted beetle that appeared in a newly started culture of the black beetle (Figure 1). Given the broad ecological overlap of our unknown beetle types with *H. trifurcata*, and the high frequency of color polymorphisms in coccinellids, we initially hypothesized that the black and spotted beetles were a regional color polymorphism of *H. trifurcata* that had been overlooked. Alternatively, we speculated the black beetles could be *Hyperaspis simulans* Casey. Gordon (1985) said of *H simulans*: “The regularly oval form and nearly black, immaculate appearance characterize *H. simulans* externally, and enable it to be separated from other southwestern species of *Hyperaspis*.” Some specimens labeled as *H. simulans* in the University of Arizona Insect Collection (UAIC) had been collected from cactus with cochineal and appeared very similar to the black beetles in our culture. We asked the following questions: (1) Are the black and spotted coccinellids color polymorphisms of *H. trifurcata*, or are they one or two separate species? (2) If the black and spotted beetles are a single separate species, are they two forms of *H. simulans*? We used morphological (male genitalia) data, molecular phylogenetics, and crossing tests to establish the relationship among the three beetles, and to reveal that the black and spotted beetles are the same species and share mitochondrial COI haplotypes, but are neither *H. simulans* nor *H. trifurcata*, but instead appear to be an undescribed species. In additional crosses, we sought to answer one other question: (3) What is the inheritance pattern of the rare spotted phenotype?

Second, we show that an unusual‐looking flightless ant‐mimic parasitoid wasp, *Formicencyrtus thoreauini*, is a primary parasitoid of *D. opuntiae*. Although the type specimen of *F. thoreauini* is recorded as emerging from *Coccus confusus* (=*Dactylopius confusus*) (Girault, 1916) and other museum and host records also record this wasp as being associated with cochineal, it was unclear whether the scale insects or coccinellid beetle larvae were the true hosts. One could easily be misled since beetle larvae often hide within the cochineal wax, perhaps to avoid another ant‐mimic encyrtid, *Homalotylus cockerelli* Timberlake, that has been shown to be a parasitoid of *H. trifurcata* (Vanegas‐Rico et al., 2015). Our initial, incorrect assumption was that *F. thoreauini* was a beetle parasitoid since we could find no unambiguous records in the literature of parasitism of cochineal, and common knowledge maintains that there are no cochineal parasitoids (e.g., Mendel et al., 2020).

## 2 METHODS

### 2.1 The coccinellid beetle, *Hyperaspis* sp.

#### 2.1.1 Beetle collection and culture

We reared *H. trifurcata* and the black coccinellid that were later shown to be a distinct species in a walk‐in rearing room (27°C, 16L:8D). Healthy, mature pads of spineless *O. ficus‐indica* were collected from the field, thoroughly washed and dried, and then infested with crawlers of *D. opuntiae* in plastic, screened boxes (381 mm × 254 mm × 89 mm). When the cochineal matured, adult beetles were introduced, pupae and adult beetles were harvested from the boxes and held in screened plastic (532 ml) cups containing crumpled laboratory cleaning tissues. The beetles were supplied with water and brewer's yeast mixed with honey.

#### 2.1.2 Crossing test methods

Experimental beetles were collected and isolated as pupae in 1.2 ml vials plugged with cotton and containing a drop of brewer's yeast and honey mixture. After emergence, the beetles were sexed (male *Hyperaspis* have white on the face) and paired; crosses to determine the compatibility of *H. trifurcata* and the black and spotted beetles were performed with single pairs on small pieces of *D. opuntiae‐*infested *O. ficus‐indica* in 270 ml cups with screened lids. Compatibility was assessed by the presence of larval offspring.

The spotted phenotype of *Hyperaspis* sp. appeared among the first progeny of a culture started from black individuals collected in the field in Tucson, AZ. Once observed, spotted beetles were segregated into a separate culture. The progeny of these spotted individuals was isolated as pupae, and only spotted parents were used to produce the next generation. For approximately five additional generations, both cultures were observed, and any individuals that differed from the expected phenotype (e.g., “black” in a “spotted” culture or vice versa) were removed. In this way, we strove to increase the homozygosity of color loci prior to performing crosses to investigate the genetic basis for color. We investigated color morph inheritance with a black X spotted cross, a cross of F1 progeny, and a backcross of black males with F1 females. For these crosses, larger‐screened containers (241 × 171 × 63 mm) and pieces of cactus pads (at least 80 × 100 mm) were used. Larval offspring were transferred to a fresh pad to complete their development when the cactus piece rotted. For backcrosses, black males drawn from the colony were crossed with isolated virgin F1 females. Most F1 crosses involved isolated individuals, but in a few instances, F1 females which had not been isolated were used for crosses with siblings. For both the F1 × F1 cross and the backcross, results were compiled from single pairs and a single cross of five females and five males. Pupae were collected from each of the crosses, and the number of each phenotype was recorded after adults eclosed. We counted progeny and compared progeny numbers to ratio predictions from Mendelian genetics.

#### 2.1.3 Morphological analysis of male genitalia

Specimens from the lab cultures were compared to *Hyperaspis* in the University of Arizona Insect Collection (UAIC) and Gordon (1985) to verify species identification.

Based on external morphology, the lab specimens most closely matched several series of specimens identified as *H. simulans*. These included a series collected in Tucson (May 1956) “assoc. cochineal on cholla cactus,” a series collected in Tucson (1960) associated with “cochineal on Christmas cholla,” and a series collected in Phoenix (1930) “taken on cochineal on cactus.” None of these series included a spotted morph like those found in laboratory cultures (Figure 1c,f). However, the detailed *H. simulans* species description of Gordon (1985) did not mention cochineal or cactus, and we wondered whether these specimens could have been misidentified.

To further explore species identification, male reproductive structures (from lab‐cultured specimens) were examined. Specimens were cleared in cold 10% KOH, rinsed in water, and then passed through progressions of EtOH up to 100% to stop the clearing process. Specimens were disarticulated, slide mounted, and examined using a compound microscope to view genitalia and other morphological characters.

#### 2.1.4 Molecular phylogenetics and curation of specimens

DNA extractions of beetles were performed with both non‐destructive (for later curation of the specimen) and destructive methods. Extractions were performed on *Hyperaspis* collected in Tucson, AZ, USA, and on laboratory culture specimens, all preserved in 95% EtOH or fresh frozen. Those from ethanol were first rinsed and soaked in water prior to extraction. Initial non‐destructive DNA extractions of the beetles involved removing one or two legs, crushing them in a tube with 5 μl of proteinase K (20 mg/ml) and 50 μl of 5%–10% Chelex in water, followed by overnight incubation at 56°C, and a final 8 min incubation at 96°C to inactivate the proteinase K. This method often did not yield sufficient DNA for amplification, so the abdomens of subsequent specimens were breached and the whole insect was incubated overnight in lysis buffer, followed by standard extraction methods for the Qiagen DNeasy Blood and Tissue kit. Following extraction, the beetles were transferred to 95% ethanol for preservation in the UAIC. Additional destructive extractions were performed using the Qiagen Blood and Tissue Kit.

*Hyperaspis* sp. COI was amplified with LCO1490 (GGTCAACAAATCATAAAGATATTGG) /HCO2198 (CCTTGGGTGGGTTGTTCTT) primers (Folmer et al., 1994) using a 53°C annealing temperature in 30 μl reactions including 2.4 μl of 10 mM dNTPs, 3 μl of each 5 μM primer, 0.18 μl Taq, and 5 μl DNA. *Hyperaspis trifurcata* COI did not amplify well with those primers but was more reliably amplified with the degenerate primers, LCO1490_puc (TTTCAACWAATCATAAAGATATTGG)/HCO2198_puc (TAAACTTCWGGRTGWCCAAARAATCA) (Talamas et al., 2019).

PCR products were quantified, normalized, and sequenced in forward and reverse directions using Sanger sequencing methods at Eton Biosciences or the University of Arizona Genetics Core (UAGC) using an Applied Biosystems 3730 DNA Analyzer (Thermo Fisher Scientific). Chromatograms were assembled into contigs, and initial base calls were made using Phred (Green & Ewing, 2002) & Phrap (Green, 1999) as implemented by the Chromaseq 1.52 (Maddison & Maddison, 2020) module within Mesquite 3.7 (Maddison & Maddison, 2021). Final base calls were made through visual inspection of the contigs. All sequences were submitted to BOLD and GenBank (Table 3).

For the phylogenetic analysis, all publicly available sequences of the 5′ regions of cytochrome c oxidase subunit I (COI‐5P) for *Hyperaspis*, and its sister group *Diomus* (Seago et al., 2011), were downloaded from the Barcode of Life Database (BOLD) on October 5, 2021. Sequences were aligned in MAFFT 7.49 (Katoh, 2013) as orchestrated by Mesquite 3.70 (Maddison & Maddison, 2021). Codon positions were inferred by minimizing the number of stop codons in the alignment while using the “Invertebrate Mitochondrial” genetic code. The matrix was trimmed to include only the 5′ region of COI. After trimming, sequences that were at least 500 base pairs long and the sequences obtained specifically for this study were included in the phylogenetic analysis (Table 3). The final matrix was trimmed to remove incomplete terminal codons and was initially partitioned by codon position. Best partition schemes and substitution models (‐TESTMERGE), tree topology, and bootstrap support values (1000 ultrafast bootstrap replicates) were performed under maximum likelihood (ML) in IQTREE v2.1.2 (Nguyen et al., 2015) on the CIPRES Science Gateway (Miller et al., 2010). Best partition schemes and substitution models were selected based on the lowest Bayesian information criterion (BIC) values.

### 2.2 The parasitoid wasp, *F. thoreauini*

#### 2.2.1 Wasp collection and culture

To distinguish between the hypotheses that *F. thoreauini* developed as a parasitoid of *Hyperaspis* or cochineal, field collected wasps were added to boxes with *D. opuntiae* alone as well as to boxes with *D. opuntiae* and *H. trifurcata* larvae of mixed ages. Some observations of oviposition were made, followed by dissections at various intervals after oviposition. For dissections, cochineal was removed from *Opuntia* pads, the wax around the insect was removed as much as possible, and the insect was transferred to a drop of saline solution on a microscope slide. The cochineal was dissected with fine‐tip forceps and mounted minuten pins. Images were taken with an Olympus Digital Camera mounted on either a dissecting microscope or a compound microscope.

## 3 RESULTS AND DISCUSSION

### 3.1 Distinguishing the three coccinellid phenotypes

In crossing tests among beetles with the characteristic striped *H. trifurcata* phenotype and black and spotted phenotypes, black or spotted beetles paired with *H. trifurcata* produced no larval progeny (Table 1). This result indicates that black and spotted phenotypes are not *H. trifurcata* color morphs but are distinct species. Further, black and spotted beetles were interfertile (Table 1), suggesting they are color morphs of a single species.

**TABLE 1:** Species limits crosses among “black,” “spotted,” and *H. trifurcata* adults. Adults were paired singly or in groups of five females and four to five males in arenas with a portion of a *O. ficus‐indica* pad infested with *D. opuntiae.* Data presented are the number of crosses in which larvae were produced/total number of crosses performed. No progeny was produced from crosses between *H. trifurcata* and either “black” or “spotted” phenotypes, but “spotted” and “black” were interfertile. As part of the current study, “black” and “spotted” phenotypes were identified as color morphs of *Hyperaspis* sp.

Phenotype	*H. trifurcata* ♀♀	“Black” ♀♀	“Spotted” ♀♀
*H. trifurcata* ♂♂	5/6	0/6	0/3
“Black” ♂♂	0/5	7/7	12/12
“Spotted” ♂♂	0/3	4/4	16/16

After study and consultation with an expert in this group, the shape of the male genitalia indicated that the black and spotted morph beetles were not *H. simulans* (N. Vandenberg, USDA Systematics Entomology Laboratory, personal communication), or did the genitalia and general appearance match any other described species in the Gordon (1985) monograph, but appear to be a member of the *conclusa* group of the genus *Hyperaspis*, known from Argentina, Chile, Bolivia, and French Guiana (N. Vandenberg, personal communication).

### 3.2 *Hyperaspis* sp.

**FIGURE 2:**
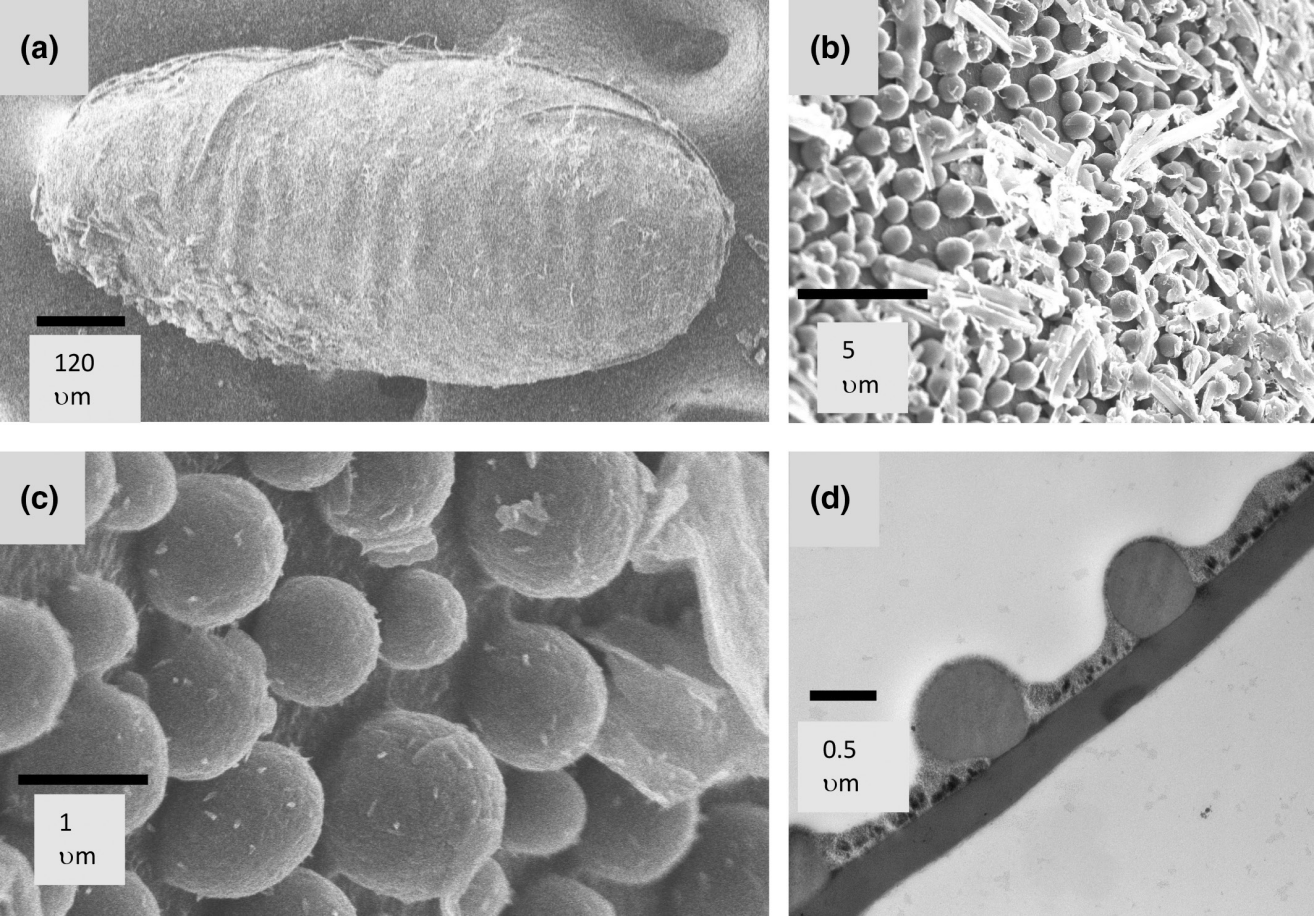
Electron micrographs of eggs of *H. trifurcata; Hyperaspis* sp. eggs are similar. (a) Scanning electron micrograph (SEM) of the entire *H. trifurcata* egg. (b and c) Higher magnification SEM shows a dense covering of small spheres on the egg surface. (d) Transmission electron micrography of egg chorion with egg interior at the bottom shows the homogenous nature of the external spheres. The function of this droplet‐like deposit is unknown.

In the current study, *Hyperaspis* sp. immature stages differed from *H. trifurcata* after the egg stage. Eggs of both species were similar (Figure 2), and interestingly, scanning electronic micrographs showed that the egg surface is coated with a layer of spheres or droplets, each about 1 μm in diameter. This layer was absent in mature ovarian eggs dissected from females, suggesting it was added during oviposition. A transmission electron micrograph cross‐section of several spheres shows them to be homogenous and not membrane bound, perhaps suggesting they are applied as droplets of a fluid (Figure 2d). The function of this layer is unclear, but one possibility is that the substance could help the egg adhere to the cochineal wax, or perhaps contribute to anti‐predator chemical disguise or defense. Beetle eggs are generally laid within the cochineal wax, and observations in the laboratory suggest the newly hatched larvae, which have been observed feeding on crawlers of the cochineal near the mature female, may remain hidden within the wax of a single individual or cluster of cochineal for several days, before eventually moving to another individual or cluster. Larval movements can sometimes be discerned by trails of bright red fecal droplets left on the cactus surface.

**FIGURE 3:**
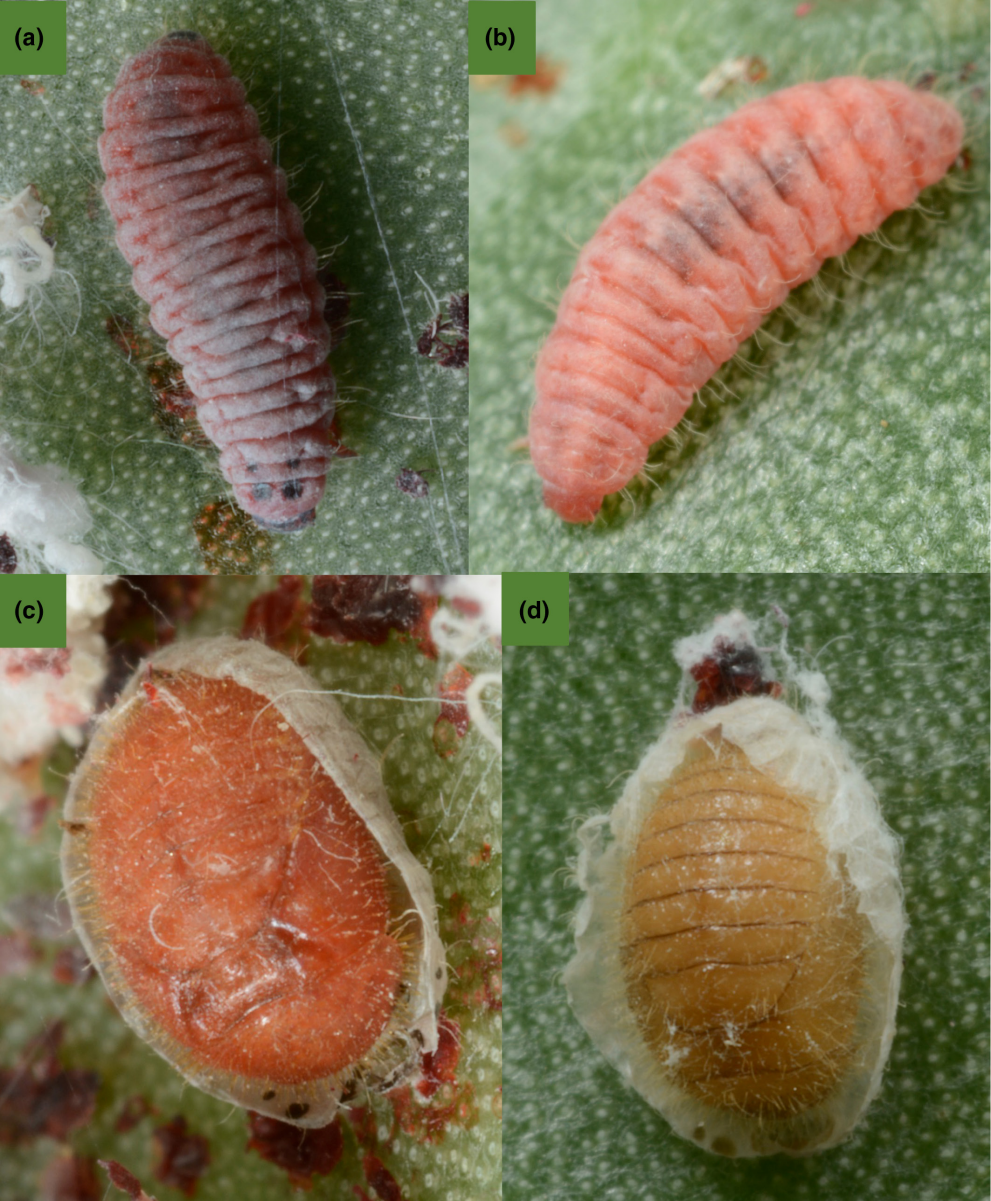
*Hyperaspis trifurcata* larva (a) and *Hyperaspis* sp. larva (b) and pupa (c) and pupa (d). All images are oriented with the head down. The beetles pupate within the split exuvium of the last larval instar, and the exuvium and the pupa are anchored to the cactus pad by a dried fecal plug that can be seen at the top of (d). The two species' immature stages differ in characteristic color.

Larvae and pupae of the two species differ in appearance. *H. trifurcata* larvae are dark maroon and have two pairs of black dorsal spots near the head (Figure 3a), while *Hyperaspis* sp. larvae are a brighter shade of red‐orange and are missing the dorsal spots (Figure 3b). Both species of beetle pupate within the split papery remnants of their last larval exuvium (Figure 3c,d), stuck to the cactus pad by a red fecal plug. *Hyperaspis trifurcata* pupae are also a deeper color than *Hyperaspis* sp. pupae (Figure 3c,d). Adults of both species eclose within the pupal sheath and often remain motionless there for a few days before venturing out. Adults of both species vary in size, and while there is overlap, *Hyperaspis* sp. is, on average, smaller (Figure 1).

**FIGURE 4:**
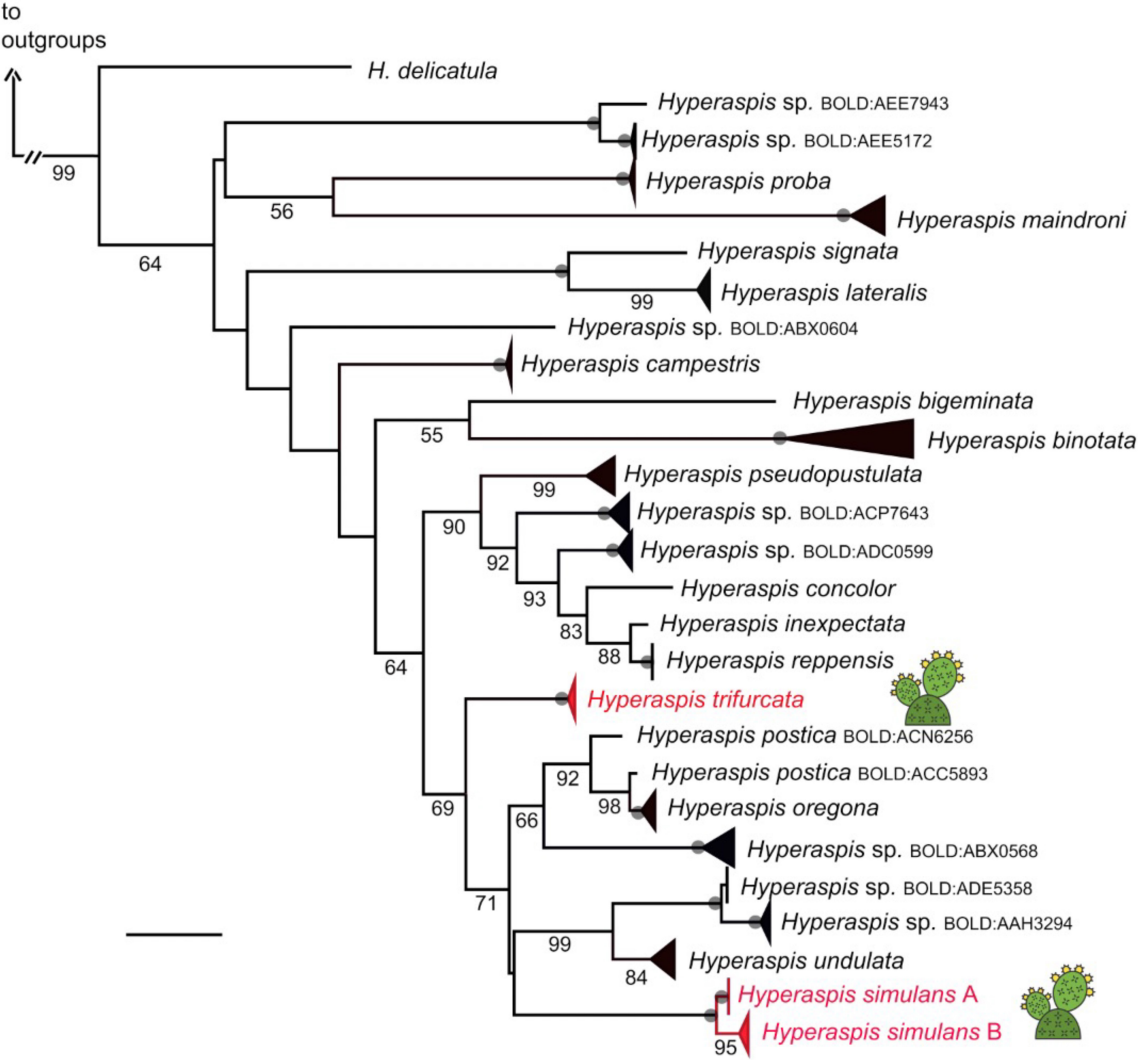
Maximum‐likelihood tree of *Hyperaspis* species based on COI. Branch length is shown proportional to relative divergence, as estimated by IQ‐TREE; scale bar indicates 0.04 units. Bootstrap support values of 100 are indicated by the gray dots on the nodes, values between 50 and 99 are below branches. Outgroups are not shown. The two species of *Hyperaspis* found in association with cochineal in this study are in red font and are indicated with the cactus icon to their right. Both clades of *Hyperaspis* sp. contain the black and the spotted morphs.

The molecular phylogeny of COI sequences confirms the results of crossing tests and morphological analysis (Figure 4), confirming the value of molecular barcoding for uncovering cryptic diversity (Bickford et al., 2007). *Hyperaspis* sp. consists of two closely related haplotypes, and black and spotted forms were found in both clades. Although *H. trifurcata* and *Hyperaspis* sp. appear embedded in one clade of *Hyperaspis*, they are not one another's closest relatives (Figure 4).

It is puzzling that two *Hyperaspis* species of similar size appear to occupy the same niche on southern Arizona cochineal, in apparent conflict with the competitive exclusion principle (Gause, 1934; Hardin, 1960). It is not uncommon to see both beetles on the same cactus or to even find them next to one another or clustered in the same crevices. We cannot say whether either species occupies additional habitats or attacks alternative prey. However, at least *Hyperaspis trifurcata* was confirmed to be a specialist prior to being introduced to Israel for biological control (Mendel et al., 2020). More likely, spatial and/or temporal niche partitioning could explain the two beetles' persistence (Amarasekare, 2003). Cactus and cochineal are patchily distributed in both urban and desert landscapes, and our observations suggest that not all predators and parasitoids are found in all patches with cochineal. We have casually observed cochineal undergoing large fluctuations in abundance in a patch over time, reduced by predation and precipitation, and perhaps promoted by dry weather, predator parasitism, and ant protection. As one possible means of coexistence, if one beetle species prevails in interspecific competition within patches, the other could persist by greater dispersal, a type of spatial niche partitioning (Amarasekare, 2003).

At least two other species of *Hyperaspis* have been observed to feed on cochineal in the USA: *Hyperaspis cruenta* LeConte (Hunter et al., 1912) and *Hyperaspis significans* Casey (Dobzhansky, 1941). Several other species of *Hyperaspis* have been shown to feed on *Dactylopius opuntiae* in Northern Africa and the Middle East, where cochineal are not native, but whether these opportunistic predators are able to reproduce on cochineal has not been determined (Bouharroud et al., 2019).

Crossing tests designed to investigate the genetic basis of the spotted color morph of *Hyperaspis* sp. conformed well to the predictions for a single‐locus, autosomal‐dominant trait (Table 2). All the progeny of the parental cross were spotted, approximately three‐fourths of the progeny of the F1 cross were spotted, and half of the progeny of the backcross between F1 females and black males were spotted. Coccinellid color polymorphisms have been investigated in a few common species after pioneering work in *Harmonia axyridis* (Pallas) by Dobzhansky (Dobzhansky, 1924). Generally, the color pattern is determined by a few genes (Ando & Niimi, 2019; Majerus, 1994, 2016). Where a single‐locus inheritance pattern is found, the gene could be a transcription factor, or a “supergene,” a cluster of genes that are tightly linked, in some cases by inversions, and inherited as if a single locus (Thompson & Jiggins, 2014). Similarly, the inheritance of color variants of *Hyperaspis significans*, one with marginal spots and a less common all‐black variant, was hypothesized to be due to a single gene, since no intermediate phenotypes were observed (Dobzhansky, 1941).

**TABLE 2:** Exploration of the inheritance pattern of the spotted phenotype in *Hyperaspis* sp. Before performing the crosses listed here, the black and spotted phenotype beetles were reared in separate cultures for five generations, isolating the pupae and removing the alternative phenotype each generation to try and ensure homozygous parents. “Expected” ratios are those predicted if “spotted” is a single‐locus autosomal‐dominant trait.

Cross‐type	Total spotted progeny	Total black progeny	Ratio spotted: black	Expected ratios
Parental black × spotted (*n* = 5)	75	0	100: 0	100: 0
F1 × F1 (*n* = 11)	95	30	76: 24	75: 25
Backcross (F1 × black) (*n* = 8)	64	55	53.8: 46.2	50: 50

Although not sister taxa, the adult *Hyperaspis* sp. bearing spots were similar in appearance to some color variants seen in *H. trifurcata*, with less pronounced stripes of cream‐colored pigment than in typical *H. trifurcata* (Figure 1). Indeed, we believed one of our wild‐caught specimens to be a spotted morph of *Hyperaspis* sp. until sequencing of CO1 demonstrated it to be *H. trifurcata*, underscoring the value of sequencing for species delineation. It is clear that the rare spotted color morph pattern is a variation on a basic *Hyperaspis* pattern as hypothesized by Dobhzansky (Dobzhansky, 1941). This ground plan includes five potential spots which can be present or absent and may fuse or vary in shape and exact location according to the species (Dobzhansky, 1941). *Hyperaspis trifurcata* most often has all five spots merged into a continuous vitta or stripe extending down from the discal spot, curving around the margin of the elytra (Figure 1), and ending at the basal spot, but some individuals have the basal or discal spots isolated, reduced, or missing. There are common elements in the color pattern within the clade containing both *Hyperaspis* sp. and *H. trifurcata*, all of which have some cream color on their elytra or pronotum*. Hyperaspis postica* LeConte, for example, has a large apical spot at the posterior tip of the elytra where both spotted *Hyperaspis* sp. and *H. trifurcata* have color, and *H. undulata* (Say) has a lateral stripe, as do *H. trifurcata* and spotted *H*yperaspis sp. *Hyperaspis undulata* also has prominent discal spots on the elytra in the area where the *H. trifurcata* stripe resumes after interruption near the elytron center. All four (*H. trifurcata*, *H. undulata*, *H. postica*, and spotted and black male *Hyperaspis* sp.) have a stripe on the outer edge of the pronotum.

While genetic constraints on color forms in this clade are likely, we cannot rule out the possibility that beetles converge on similar phenotypes for Müllerian mimicry as well since *Hyperaspis* species have chemical defenses that are likely to make them distasteful. Eisner et al. (1994) showed that the carmine in the cochineal prey of *H. trifurcata* was acquired by the beetle and was distasteful to ants. The Old World scale predator *Hyperaspis campestris* (Herbst) was found to produce “hyperaspine,” a novel bitter alkaloid, adding to a list of defensive alkaloids identified in many other coccinellids (Lebrun et al., 2001).

### 3.3 The parasitoid wasp, *F. thoreauini*

**FIGURE 5:**
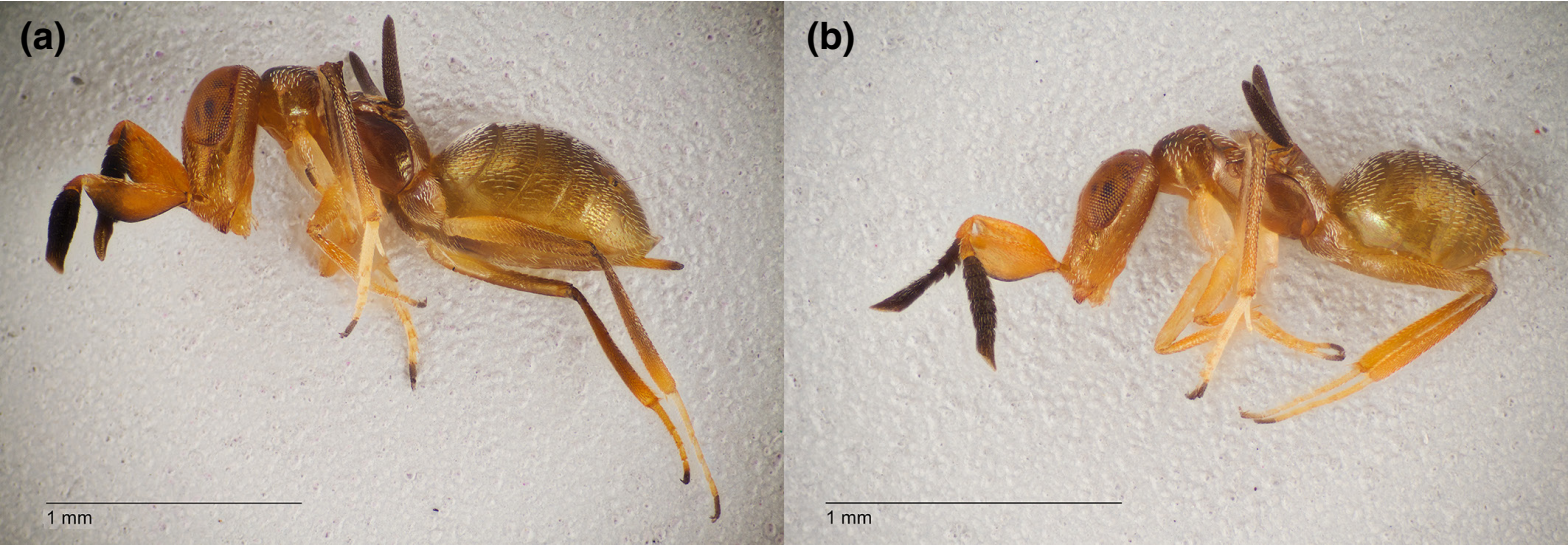
*Formicencyrtus thoreauini* (Encyrtidae), (a) female and (b) male. Our results indicated that this wasp is a primary parasitoid of *Dactylopius opuntiae*, a cochineal insect.

**FIGURE 6:**
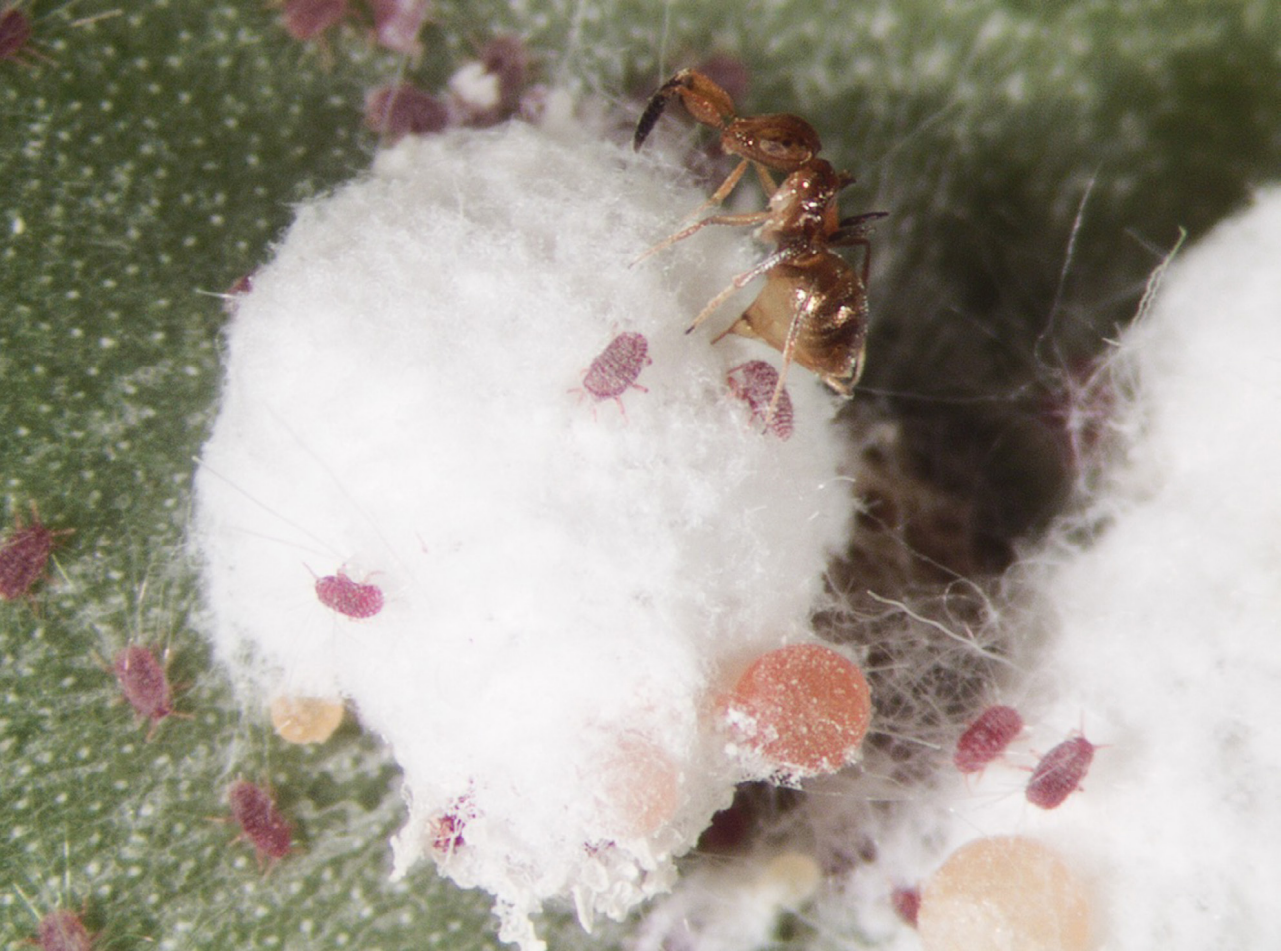
*Formicencyrtus thoreauini* female ovipositing through the wax covering over a *Dactylopius opuntiae* cochineal. Because the coccinellid beetle eggs and larvae typically hide within the cochineal wax, the host of this wasp was not initially clear. First instar cochineal (“crawlers”) are visible in and around the wax, as are drops of accumulated honeydew excreted by the cochineal at the bottom of the image.

While we initially hypothesized that *F. thoreauini* (Figure 5) was a parasitoid of *Hyperaspis* immatures, we found no support for this hypothesis, and instead our results indicate that *F. thoreauini* is a parasitoid of *D. opuntiae*. We observed females ovipositing into wax containing cochineal (Figure 6), and dissections of cochineal in pure culture of *F. thoreauini* and *D. opuntiae* showed eggs and larvae of various stages. Additionally, we successfully reared two consecutive generations of *F. thoreauini* from a pure culture of *D. opuntiae* in the laboratory. Between one and four wasps emerged from the mummies that resulted. From these results, we can categorize *F. thoreauini* as a semi‐gregarious wasp, with clutches of one to four or five, and a primary parasitoid of at least *D. opuntiae*. The type specimen was recorded as being from *Coccus confusus* (=*D. confusus*) (Girault, 1916). Further, when we presented *F. thoreauini* with both *D. opuntiae* and *Hyperaspis* larvae, no beetles became parasitized. In contrast, field collections of infested cactus pads regularly yielded mummified *Hyperaspis* larvae from which emerged the gregarious beetle parasitoid *Homalotylus cockerelli*.

**FIGURE 7:**
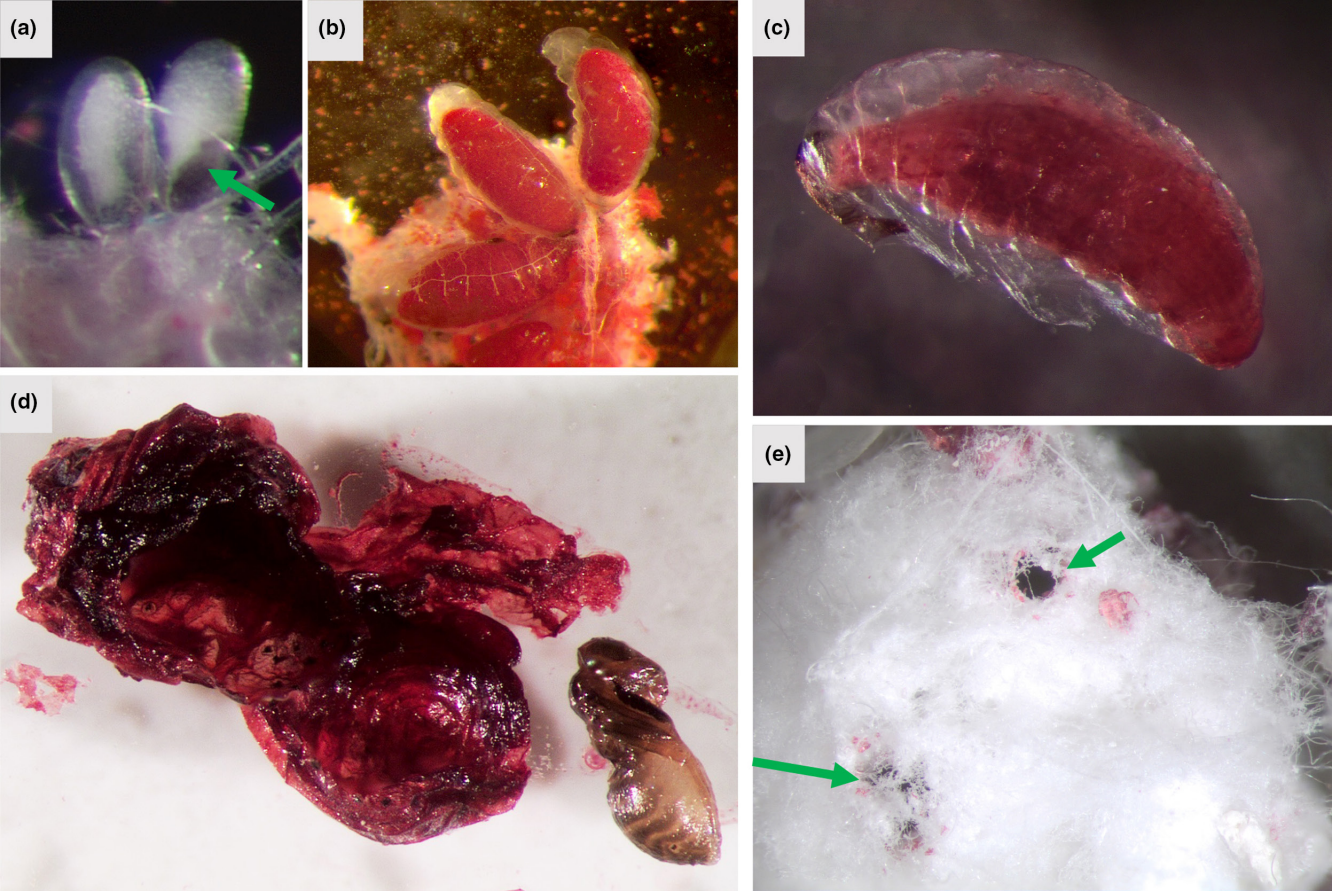
Stages of *Formicencyrtus thoreauini*. (a) Eggs dissected from *Dactylopius opuntiae*, attached to host tissue at the posterior end. Arrow points to the dark gray aeroscopic plate. (b) Early instar larvae, attached in the tail region to host tissue. (c) Later instar larva, enclosed in a loose membrane. (d) Pupa dissected from desiccated cochineal mummy following removal of external wax. The pupa was found in a smooth‐walled, gas‐filled chamber within the desiccated cochineal remains. (e) Holes in the cochineal made by eclosing *F. thoreauini* (arrows) are often obscured by wax.

Prinsloo (1997) provides an excellent review of various types of encyrtid parasitoid development within soft scales which, along with a detailed description of the development of *Encyrtus saliens* Prinsloo & Annecke provided by Wright (1986), we used to help explain *F. thoreauini* development. Dissections of *F. thoreauini* females showed the ovarian eggs to be dumbbell shaped, with a neck separating two bulbs. This extended chorion envelope is known to allow the egg to pass through the ovipositor more easily, after which the contents flow into the posterior end, the location of the embryo (Figure 7a; Prinsloo, 1997). *Formicencyrtus thoreauini* eggs were of the banded type, in which sculpturing of the neck between the bulbs forms an aeroscopic plate. The aeroscopic plate allows gas exchange between the outside of the host, where the collapsed anterior end of the egg forms a tab penetrating the host integument and the embryo. In this type of development, the first instar larva remains attached to the aeroscopic plate and thus the host integument, with its posterior end enclosed within the egg chorion. Prinsloo (1997) describes this type of development as metapneustic (with one or two pairs of caudal spiracles present in early instar larvae). Wright (1986) demonstrated attachment of the larva to the aeroscopic plate via caudal tracheal extensions well into the fifth instar of *Encyrtus saliens*. While we could not detect egg tabs on the outside of the highly corrugated and wax‐covered cuticle of cochineal, we did observe the site of attachment on the interior of the cochineal cuticle in dissected specimens and were able to confirm a connection between the aeroscopic plate and the cuticle. We also saw evidence of at least two larval exuviae remaining attached after pupation and/or emergence of the wasps.

Later instar encyrtid larvae may produce a membrane within which they continue development, and which becomes connected to host tracheae that permit gas exchange with the late/final instar larva and pupa within the membrane (Prinsloo, 1997; Wright, 1986). In *F. thoreauini*, a loose membrane was visible surrounding later instars (Figure 7c), although we cannot be sure of the membrane source, or of host tracheal attachment. Further, each larva appears to pupate in a dry, gas‐filled compartment within the cochineal host, likely the remnants of the membrane segregating larva and host hemolymph (Figure 7d). This adaptation allows a single wasp to successfully complete its development in a mature cochineal, which is not entirely consumed by the larva before pupation (Figure 7d). When multiple *F. thoreauini* develop in the same host, each wasp pupates within its own membrane and the entire cochineal is consumed. Smaller‐ or variable‐sized adults have been noted when three or more wasps emerged from a single host. Eclosing adults then chew out of the cochineal mummy, leaving often inconspicuous holes in the cochineal wax (Figure 7e). In our laboratory, *F. thoreauini* development was relatively slow, about 25 days to adulthood at 27°C, but they may develop more quickly in the warmer temperatures of their desert habitat.

While the host of *F. thoreauini* was revealed by the current study, several questions remain about this species. What is the function of the highly modified wings which appear as spikes on the thorax of the adults? It is tempting to imagine that they may excrete compounds, perhaps for ant appeasement. In informal observations in the laboratory, ants added to the rearing box did not appear to bother these wasps as they did beetles, but it is unclear whether natural behavior was being observed in the highly artificial conditions of the laboratory. Second, how do these micropterous wasps travel between patches of cochineal on cactus, surrounded as they are by inhospitable hot and dry desert soils? In our observations and laboratory cultures, both males and females are micropterous. Fully winged individuals collected in California were provisionally assigned to this species but have yet to be confirmed and likely represent another species (Zuparko, 2015; R. Zuparko, Essig Museum of Entomology, University of California, Berkeley, personal communication). If *F. thoreauini* are entirely micropterous, we do not easily understand the dispersal of this species. While we have observed these wasps to be excellent jumpers, jumping would seem to be of limited value to travel between patches. If they are phoretic, it is not clear what larger animal would serve as a dependable source of transmission to another patch of habitat, although jumping might be valuable for jumping on or off a larger animal.

Because some cochineal species are exotic pests in areas where exotic *Opuntia* species have economic value, a parasitoid of *D. opuntiae* may be of interest for ecologists considering biological control of cochineal, especially if *F. thoreauini* has a more limited host range than do many of the cochineal predators. *Formicencyrtus thoreauini* might be an effective biological control agent in the absence of competing predators. There are aspects of the biology of *F. thoreauini* that might limit this species as a biological control agent, however. This wasp has slow host‐handling and oviposition rates, and a long development time. It is also flightless and unlikely to spread rapidly, usually a disadvantage in a natural enemy. However, this species' limited mobility may make it a better candidate for biological control in local areas, given the geographic conflicts of interest in cochineal biological control.

To conclude, we found two uncharacterized natural enemies of *D. opuntiae*, a common and conspicuous insect in the neighborhoods of Tucson, AZ, USA, to be hiding in plain sight, within a short walk of the University of Arizona, and within a human community of many active naturalists. These findings underscore that discovery awaits an engaged observer even in apparently well‐studied communities. The geographic range of *Hyperaspis* sp. is still to be determined. *Formicencyrtus thoreauini* was described from specimens collected in New Mexico (Girault, 1916) and is found in Arizona and possibly California (Zuparko, 2015). Neither is described in publications characterizing the communities in Texas (Gilreath & Smith, 1988) nor central Mexico (Vanegas‐Rico et al., 2010). How these two community members interact with other natural enemies, cochineal‐tending ants, and the many parasitoids and hyperparasitoids in the cochineal community await further exploration.

## AUTHOR CONTRIBUTIONS

**Suzanne E. Kelly:** Conceptualization (lead); data curation (lead); formal analysis (supporting); investigation (lead); methodology (lead); writing – review and editing (equal). **Wendy Moore:** Data curation (supporting); formal analysis (lead); methodology (lead); supervision (equal); visualization (lead); writing – review and editing (equal). **W. Eugene Hall:** Data curation (supporting); formal analysis (supporting); investigation (equal); methodology (lead); writing – review and editing (equal). **Martha S. Hunter:** Funding acquisition (lead); project administration (lead); supervision (lead); writing – original draft (lead); writing – review and editing (equal).

## CONFLICT OF INTEREST

The authors declare no conflict of interest.

## Data Availability

all data (sequence information as well as sample metadata) can be accessed at BOLD and GenBank (see Table 3 for accession numbers). Taxon sampling table and accession numbers for sequences generated specifically for this study. For *Hyperaspis* sp., we note specimen color morph and clade membership as depicted in Figure 4.

## References

[ece39151-bib-0001] Amarasekare, P. (2003). Competitive coexistence in spatially structured environments: A synthesis. Ecology Letters, 6(12), 1109–1122. 10.1046/j.1461-0248.2003.00530.x

[ece39151-bib-0002] Anderson, N. O. , & Olsen, R. T. (2015). A vast array of beauty: The accomplishments of the father of American ornamental breeding, Luther Burbank. HortScience, 50(2), 161–188. 10.21273/hortsci.50.2.161

[ece39151-bib-0003] Ando, T. , & Niimi, T. (2019). Development and evolution of color patterns in ladybird beetles: A case study in *Harmonia axyridis* . Development, Growth and Differentiation, 61, 73–84. 10.1111/dgd.12592 30644547

[ece39151-bib-0004] Annecke, D. , & Moran, V. (1978). Critical review of biological pest control in South Africa, II. The prickly pear, *Opuntia ficus‐indica* (L.) Miller. Journal of the Entomological Society of South Africa, 41, 161–188.

[ece39151-bib-0005] Badii, M. H. , & Flores, A. E. (2001). Prickly pear cacti pests and their control in Mexico. Florida Entomologist, 84(4), 503–505. 10.2307/3496379

[ece39151-bib-0006] Bickford, D. , Lohman, D. J. , Sodhi, N. S. , Ng, P. K. L. , Meier, R. , Winker, K. , Ingram, K. K. , & Das, I. (2007). Cryptic species as a window on diversity and conservation. Trends in Ecology & Evolution, 22(3), 148–155. 10.1016/j.tree.2006.11.004 17129636

[ece39151-bib-0007] Bouharroud, R. , El Aalaoui, M. , Boujghagh, M. , Hilali, L. , El Bouhssini, M. , & Sbaghi, M. (2019). New record and predatory activity of *Hyperaspis campestris* (Herbst 1783) (Coleoptera: Coccinellidae) on *Dactylopius opuntiae* (Hemiptera: Dactylopiidae) in Morocco. Entomological News, 128(2), 156–160. 10.3157/021.128.0202

[ece39151-bib-0008] Dobzhansky, T. (1924). Die geographische und individuelle Variabilitat von *Harmonia axyridis* Pallas in ihren Wechselbeziehungen [In German]. Biologische Zentralblatt, 44, 401–421.

[ece39151-bib-0009] Dobzhansky, T. (1941). Beetles of the genus *Hyperaspis* inhabiting the United States. Smithsonian Miscellaneous Collections, 101, 1–94.

[ece39151-bib-0010] Eisner, T. , Ziegler, R. , McCormick, J. L. , Eisner, M. , Hoebeke, E. R. , & Meinwald, J. (1994). Defensive use of an acquired substance (Carminic acid) by predaceous insect larvae. Experientia, 50(6), 610–615. 10.1007/bf01921733 8020623

[ece39151-bib-0011] Folmer, O. , Black, M. , Hoeh, W. , Lutz, R. , & Vrijenhoek, R. (1994). DNA primers for amplification of mitochondrial cytochrome c oxidase subunit I from diverse metazoan invertebrates. Molecular Marine Biology and Biotechnology, 3, 294–299. 7881515

[ece39151-bib-0012] García Morales, M. , Denno, B. D. , Miller, D. R. , Miller, G. L. , Ben‐Dov, Y. , & Hardy, N. B. (2016). ScaleNet: A literature‐based model of scale insect biology and systematics. Database: The Journal of Biological Databases and Curation, 2016, bav118. 10.1093/database/bav118 26861659 PMC4747323

[ece39151-bib-0013] Gause, G. F. (1934). The struggle for coexistence. Williams and Wilkins.

[ece39151-bib-0014] Gilreath, M. E. , & Smith, J. W. (1988). Natural enemies of *Dactylopius confusus* (Homoptera, Dactylopiidae) ‐ Exclusion and subsequent impact on *Opuntia* (Cactaceae). Environmental Entomology, 17(4), 730–738.

[ece39151-bib-0015] Girault, A. A. (1916). New Encyrtidae from North America. Psyche, 23, 41–50.

[ece39151-bib-0016] Gordon, R. D. (1985). The Coccinellidae (Coleoptera) of America North of Mexico. Journal of the New York Entomological Society, 93, 1–912.

[ece39151-bib-0017] Green, P. (1999). Phrap, version 0.990329. http://phrap.org

[ece39151-bib-0018] Green, P. , & Ewing, B. (2002). Phred, version 0.020425c. http://phrap.org

[ece39151-bib-0019] Greenfield, A. B. (2006). A perfect red: Empire, espionage, and the quest for the color of desire. Harper Perennial.

[ece39151-bib-0020] Griffith, M. P. (2004). The origins of an important cactus crop, *Opuntia ficus‐indica* (Cactaceae): New molecular evidence. American Journal of Botany, 91, 1915–1921. 21652337 10.3732/ajb.91.11.1915

[ece39151-bib-0021] Hardin, G. (1960). The competitive exclusion principle. Science, 132, 1292–1297. 10.1126/science.132.3419.96-a 14399717

[ece39151-bib-0022] Hunter, W. D. , Pratt, F. C. , & Mitchell, J. D. (1912). The principal cactus insects of the United States. U.S. Department of Agriculture Entomological Bulletin, 113, 1–71.

[ece39151-bib-0023] Katoh, S. (2013). MAFFT multiple sequence alignment software version 7: Improvements in performance and usability. Molecular Biology and Evolution, 30, 772–780. 23329690 10.1093/molbev/mst010 PMC3603318

[ece39151-bib-0024] Lebrun, B. , Braekman, J.‐C. , Daloze, D. , Kalushkov, P. , & Pasteels, J. M. (2001). Hyperaspine, a new 3‐oxaquinolizidine alkaloid from *Hyperaspis campestris* (Coleoptera: Coccinellidae). Tetrahedron Letters, 42, 4621–4623.

[ece39151-bib-0025] Maddison, D. R. , & Maddison, W. P. (Producer). (2020). Chromaseq: A mesquite package for analyzing chromatograms . Version 1.52. http://chromaseq.mesquiteproject.org

[ece39151-bib-0026] Maddison, D. R. , & Maddison, W. P. (Producer). (2021). Mesquite: A modular system for evolutionary analysis . Version 3.70. http://www.mesquiteproject.org

[ece39151-bib-0027] Majerus, M. E. N. (1994). Ladybirds. Harper Collins Publishers.

[ece39151-bib-0028] Majerus, M. E. N. (2016). In H. E. Roy & P. M. J. Brown (Eds.), A natural history of ladybird beetles. Cambridge University Press.

[ece39151-bib-0029] Mann, J. (1969). Cactus‐feeding insects and mites. United States Natural Museum Bulletin, 256, 1–158.

[ece39151-bib-0030] Mendel, Z. , Protasov, A. , Vanegas‐Rico, J. M. , Lomeli‐Flores, J. R. , Suma, P. , & Rodríguez‐Leyva, E. (2020). Classical and fortuitous biological control of the prickly pear cochineal, *Dactylopius opuntiae*, in Israel. Biological Control, 142, 104157. 10.1016/j.biocontrol.2019.104157

[ece39151-bib-0031] Miller, M. A. , Pfeiffer, W. , & Schwartz, T. (2010, 14 Nov 2010). Creating the CIPRES Science Gateway for inference of large phylogenetic trees . Paper presented at the Proceedings of the Gateway Computing Environments Workshop (GCE), New Orleans, LA.

[ece39151-bib-0032] Nguyen, L. T. , Schmidt, A. , von Haeseler, A. , & Minh, B. Q. (2015). IQ‐TREE: A fast and effective stochastic algorithm for estimating maximum likelihood phylogenies. Molecular Biology and Evolution, 32, 268–274. 10.1093/molbev/msu30 25371430 PMC4271533

[ece39151-bib-0033] Novoa, A. , Brundu, G. , Day, M. D. , Deltoro, V. , Essl, F. , Foxcroft, L. C. , Fried, G. , Kaplan, H. , Kumschick, S. , Lloyd, S. , Marchante, E. , Marchante, H. , Paterson, l. D. , Pyšek, P. , Richardson, D. M. , Witt, A. , Zimmermann, H. G. , & Wilson, J. R. U. (2019). Global actions for managing cactus invasions. Plants, 8(10), 421. 10.3390/plants8100421 PMC6843271 31623290

[ece39151-bib-0034] Paterson, I. D. , Klein, H. , Muskett, P. C. , Griffith, T. C. , Mayonde, S. , Mofokeng, K. , Mnqeta, Z. , & Venter, N. (2021). Biological control of Cactaceae in South Africa. African Entomology, 29, 713–734.

[ece39151-bib-0035] Paterson, I. D. , & Witt, A. (2022). Biological control of pest cactus and cactus pests in Africa. Acta Horticulturae, In press.

[ece39151-bib-0036] Portillo, L. (2009). Biogeography of Dactylopiidae and human factor. Acta Horticulturae, 811, 235–240.

[ece39151-bib-0037] Prinsloo, G. L. (1997). Chapter 2.3 Parasitoids: 2.3.1 Encyrtidae. In Y. Ben‐Dov & C. J. Hodgson (Eds.), Soft scale insects – Their biology, natural enemies, and control (7B) (pp. 69–109). Elsevier Science.

[ece39151-bib-0038] Seago, A. E. , Giorgi, J. A. , Li, J. , & Slipinski, A. (2011). Phylogeny, classification and evolution of ladybird beetles (Coleoptera: Coccinellidae) based on simultaneous analysis of molecular and morphological data. Molecular Phylogenetics and Evolution, 60(1), 137–151. 10.1016/j.ympev.2011.03.015 21426943

[ece39151-bib-0039] Spodek, M. , Ben‐Dov, Y. , Protasov, A. , Carvalho, C. J. , & Mendel, Z. (2013). First record of *Dactylopius opuntiae* (Cockerell) (Hemiptera: Coccoidea: Dactylopiidae) from Israel. Phytoparasitica, 42(3), 377–379. 10.1007/s12600-013-0373-2

[ece39151-bib-0040] Talamas, E. J. , Bon, M. C. , Hoelmer, K. A. , & Buffington, M. L. (2019). Molecular phylogeny of *Trissolcus* wasps (Hymenoptera, Scelionidae) associated with *Halyomorpha halys* (Hemiptera, Pentatomidae). Journal of Hymenoptera Research, 93, 201–217. 10.3897/JHR.73.39563

[ece39151-bib-0041] Thompson, M. J. , & Jiggins, C. D. (2014). Supergenes and their role in evolution. Heredity, 113(1), 1–8. 10.1038/hdy.2014.20 24642887 PMC4815649

[ece39151-bib-0042] Vanegas‐Rico, J. M. , Lomeli‐Flores, J. R. , Rodriguez‐Leyva, E. , Mora‐Aguilera, G. , & Valdez, J. M. (2010). Natural enemies of *Dactylopius opuntiae* (Cockerell) on *Opuntia ficus‐indica* (L.) Miller in Central Mexico [SP]. [Enemigos naturales de *Dactylopius opuntiae* (Cockerell) en *Opuntia ficus‐indica* (L.) Miller en el centro de Mexico]. Acta Zoologica Mexicana Nueva Serie, 26(2), 415–433.

[ece39151-bib-0043] Vanegas‐Rico, J. M. , Lomeli‐Flores, J. R. , Rodríguez‐Leyva, E. , Pérez‐Panduro, A. , González‐Hernández, H. , & Marín‐Jarillo, A. (2015). *Hyperaspis trifurcata* (Coleoptera: Coccinellidae) and its parasitoids in central Mexico. Revista Colombiana de Entomologia, 41(2), 194–199.

[ece39151-bib-0044] Witt, A. B. R. , Nunda, W. , Makale, F. , & Reynolds, K. (2020). A preliminary analysis of the costs and benefits of the biological control agent *Dactylopius opuntiae* on *Opuntia stricta* in Laikipia County, Kenya. BioControl, 65(4), 515–523. 10.1007/s10526-020-10018-x

[ece39151-bib-0045] Wright, E. J. (1986). Immature stages of *Encyrtus saliens* (Hymenoptera: Encyrtdae), an imported parasite of ice plant scales (Homoptera: Coccidae) in California. Annals of the Entomological Society of America, 79, 273–279.

[ece39151-bib-0046] Zuparko, R. L. (2015). Annotated checklist of California Encyrtidae (Hymenoptera). Zootaxa, 4017, 1–126. 26624025 10.11646/zootaxa.4017.1.1

