# Correction to “Hiding in Plain Sight: Cryptic Enemies Are Found on Cochineal (Hemiptera: Dactylopiidae), a Scale Insect of Economic and Cultural Significance”

**DOI:** 10.1002/ece3.72710

**Published:** 2025-12-12

Kelly, S. E., Moore, W., Hall, W. E., & Hunter, M. S. (2022). Hiding in Plain Sight: Cryptic Enemies Are Found on Cochineal (Hemiptera: Dactylopiidae), a Scale Insect of Economic and Cultural Significance. *Ecology and Evolution*, 12, e9151. https://doi.org/10.1002/ece3.9151.

Figure 4 in the published article is incorrect. The version presented was created before the examination of the male genitalia was completed, at a time when the authors mistakenly identified one of the species under study as *Hyperaspis simulans* rather than the as‐yet‐unnamed species (*Hyperaspis* sp.). The only difference between the accurate tree and the incorrect one is the inclusion of *Hyperaspis simulans* in place of *Hyperaspis* sp.; however, this represents a significant error that could lead to considerable confusion for the reader. The corrected figure is shown below.

**FIGURE 4:**
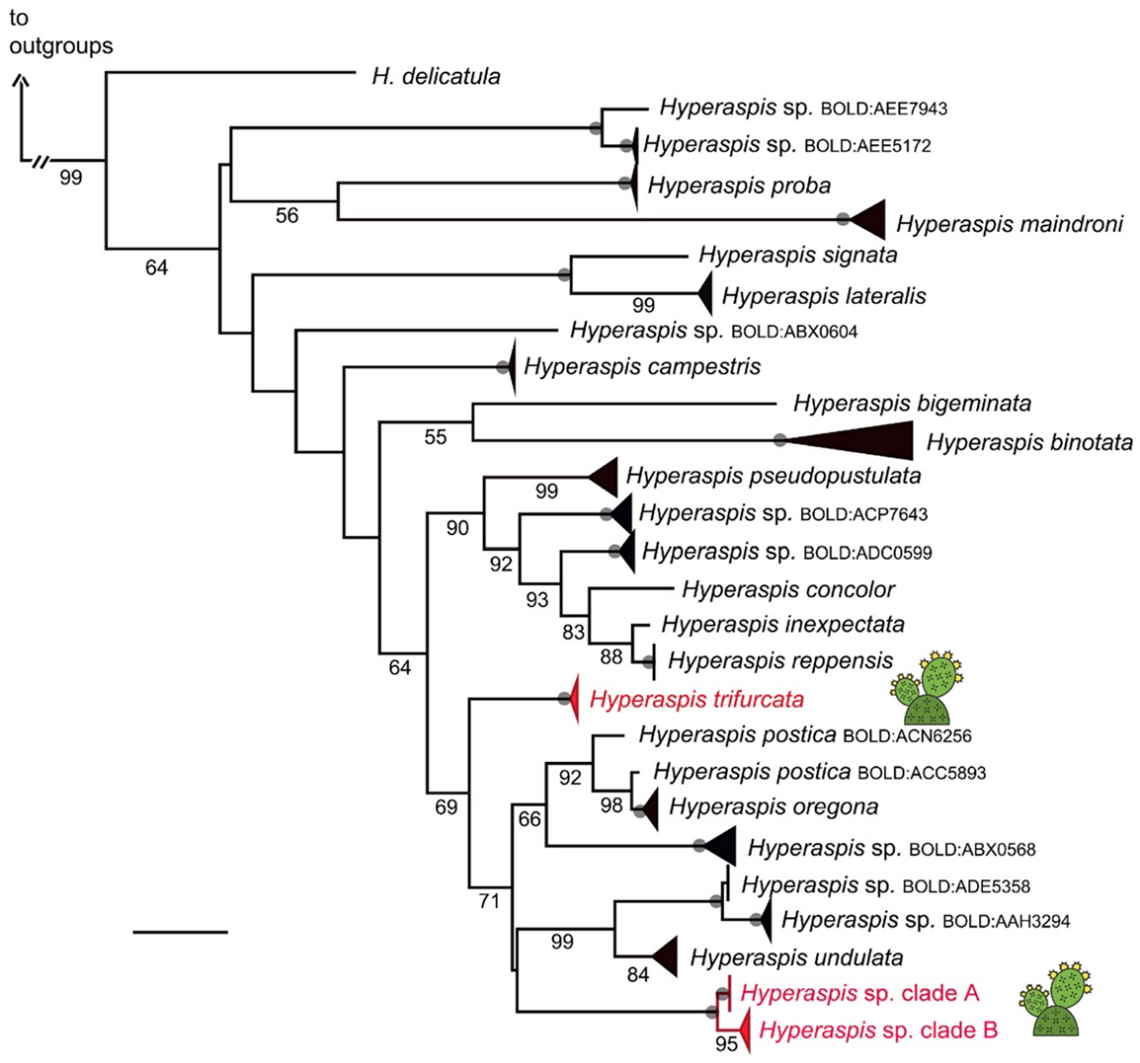
Maximum likelihood tree of *Hyperaspis* species based on COI. Branch length is shown proportional to relative divergence, as estimated by IQ‐TREE; scale bar indicates 0.04 units. Bootstrap support values of 100 are indicated by the gray dots on the nodes; values between 50 and 99 are below branches. Outgroups are not shown. The two species of *Hyperaspis* found in association with cochineal in this study are in red font and are indicated with the cactus icon to their right. Both clades of *Hyperaspis* sp. contain the black and the spotted morphs.

We apologize for this error.

